# Spontaneous duodenal fistulization of pseudocyst of pancreas: A rare entity in children

**DOI:** 10.4103/0971-9261.69141

**Published:** 2010

**Authors:** Sunil Kumar Yadav, Vipul Gupta, Ashhad Ali Khan

**Affiliations:** Department of Pediatric and Neonatal Surgery, IBN Sina Hospital, Kuwait

**Keywords:** Duodenum, pancreatic pseudocyst, spontaneous internal fistula

## Abstract

The spontaneous resolution of pseudopancreatic cyst due to internal fistulization into the duodenum in a 4-year-old male child is described here. This is rare and the child presented initially with features of duodenal obstruction. Upper gastrointestinal endoscopy and computed tomography scan confirmed the diagnosis and the unusual mode of resolution of this entity.

## INTRODUCTION

Pseudocyst of pancreas is rare in children.[1,2] A review of the literature suggests that although most cases of pseudocyst respond to conservative treatment, spontaneous resolution due to internal duodenal fistulization has scarcely been discussed in the literature.[1–7]

## CASE REPORT

**Figure 1:**
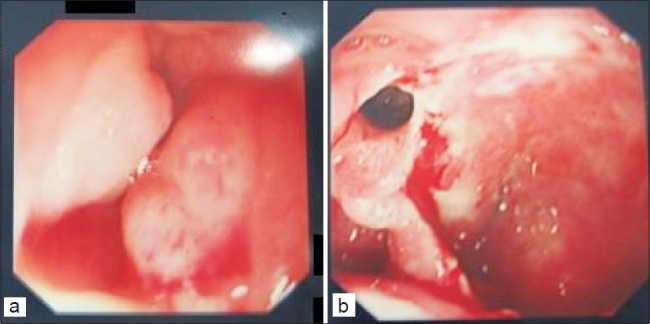
(a) Upper gastrointestinal endoscopy showing duodenal obstruction with friable duodenal mucosa; and (b) resolution of duodenal obstruction with internal fistula on duodenal wall.

A 4-year-old boy presented with 3 days history suggestive of duodenal obstruction. On clinical examination, the patient appeared irritable with no evidence of fever or hemodynamic instability. The abdominal examination was unremarkable except mild discomfort in epigastrium on palpation. Plain abdominal radiograph was suggestive of high small bowel obstruction. Upper gastrointestinal contrast study showed obstruction in the second and third part of the duodenum. The contrast did not pass beyond third part of the duodenum. He was managed conservatively with nasogastric (NG) aspiration, intravenous fluids, and H2 blockers. He had large amount of greenish aspirate from the NG tube every day. His routine hematologic investigations and biochemistry were normal except high serum amylase level (307 U/L). An abdominal ultrasound showed a cystic mass (8 × 9 cm) in the epigastrium in relation to the head of pancreas. Computed tomography scan of abdomen showed similar finding and a diagnosis of pseudocyst at the head region of pancreas was made. Upper gastrointestinal endoscopy showed narrowing of duodenal lumen beyond the second part due to extraluminal compression of C-loop of duodenum [Figure 1a]. There was an area of friable mucosa with fistula on the medial wall of the second part of duodenum in the vicinity of ampulla of Vater. A diagnosis of spontaneous internal drainage of pseudocyst of head of pancreas was thought of and no further endoscopic intervention was attempted. Conservative management was continued till the next 72 h when sudden improvement in the clinical condition was observed. His NG aspirates came down and repeat endoscopy showed complete disappearance of mass effect in duodenum with an opening in the medial wall of the second part of the duodenum suggesting spontaneous duodenal fistulization of pseudocyst of pancreas [Figure 1b]. The patient had prompt clinical recovery with resolution of symptoms. The NG tube was removed and oral feeds were resumed. He was discharged home and repeat endoscopy done after 1 month was normal. The patient is on regular follow-up for the last 2 years and is doing well.

## DISCUSSION

Although there was no definite evidence of abdominal trauma in the present case, trauma has been reported as the most common cause of pseudocyst of pancreas in children.[1–4] Features of duodenal obstruction with other nonspecific abdominal symptoms may be associated with an abdominal mass.

The natural history of pseudocyst of pancreas in children has not been well understood.[3,4] Although investigators have reported satisfactory recovery in few cases managed conservatively, operative intervention either in the form of operative or endoscopic drainage has been with satisfactory results.[4] Traditionally surgical intervention has been advised for adults with cysts more than 6 cm in size or persisting for more than 6 weeks.[5] But validity of this concept in pediatric age group is still not known. A review of the literature suggests that very few investigators have reported spontaneous resolution of pseudocyst of pancreas in 30%–60% of children along with associated complications, such as infection, rupture, or hemorrhage.[3] Surprisingly, spontaneous resolution of pseudocyst of pancreas due to internal fistulization as experienced in the present case has rarely been reported in the literature till date, although it remains a well-known phenomenon described in adults.[6] It has been described that pseudopancreatic cyst can perforate into peritoneal cavity, stomach, duodenum, colon, portal vein, pleural cavity, or through abdominal wall with etiopathogenesis been attributed to ongoing necrotic effect of pancreatic enzymes on surrounding tissues and the wall.[2] The management of internal fistulization depends on the site of intraabdominal organ involved. As seen in the present and reported cases in adults, spontaneous fistulization into stomach or duodenum can be managed conservatively, although spontaneous perforation in the peritoneal cavity or the colon may require surgical intervention so as to prevent complications, such as sepsis.[7]

Thus we conclude that pseudocyst of pancreas remains a rare entity in the pediatric age group, mostly associated with delayed diagnosis owing to its obscure etiology and nonspecific clinical presentation. Although spontaneous internal fistulization is extremely uncommon in the pediatric age group, it should be confirmed with endoscopy in pediatric patients undergoing prompt resolution of symptoms, especially when there is a large pseudocyst of pancreas.

## References

[1] Walt AJ, Bouwman DL, Weaver DW, Sachs RJ (1990). The impact of technology on management of pancreatic pseudocyst: Fifth annual Samuel Jason Mixter Lecture. Arch Surg.

[2] Pichumoni CS, Agarwal N (1999). Pancreatic pseudocyst: Which and how should drainage be performed?. Gastroenterol Clin North Am.

[3] Teh SH, Pham TH, Lee A, Stavlo PL, Hanna AM, Moir C (2006). Pancreatic pseudocyst in children: The impact of management strategies on outcome. J Pediatr Surg.

[4] Roy MK, Ralph C, Stephen W (1999). Successful Endoscopic drainage of post-traumatic Pseudocyst of Pancreas in a child. J Pediatr Surg.

[5] Warshaw Al, Rattner DW (1985). Timing of surgical drainage for pancreatic pseudocyst: Clinical and chemical criteria. Ann Surg.

[6] Lillmore K, Yeo CJ (1998). Management of complications of pancreatitis. Curr Probl Surg.

[7] Urakami A, Tsunoda T, Hayashi J, Oka Y, Mizuno M (2002). Spontaneous fistulization of pancreatic pseudocyst into colon and duodenum. Gastrointest Endosc.

